# Biomarkers in Alzheimer’s Disease Analysis by Mass Spectrometry-Based Proteomics

**DOI:** 10.3390/ijms15057865

**Published:** 2014-05-06

**Authors:** Yahui Liu, Hong Qing, Yulin Deng

**Affiliations:** School of Life Science, Beijing Institute of Technology, No. 5 Zhongguancun South Street, Haidian District, Beijing 100081, China; E-Mails: liuyahui2049@163.com (Y.L.); hqing@bit.edu.cn (H.Q.)

**Keywords:** biomarker, Alzheimer’s disease, mass spectrometry, proteomics

## Abstract

Alzheimer’s disease (AD) is a common chronic and destructive disease. The early diagnosis of AD is difficult, thus the need for clinically applicable biomarkers development is growing rapidly. There are many methods to biomarker discovery and identification. In this review, we aim to summarize Mass spectrometry (MS)-based proteomics studies on AD and discuss thoroughly the methods to identify candidate biomarkers in cerebrospinal fluid (CSF) and blood. This review will also discuss the potential research areas on biomarkers.

## 1. Introduction

A biomarker is a substance used as a measurable indicator of a particular biological stage, such as normal biological process and pathogenic process. Specifically, it is one that reveals risk characteristics, the disease’s presence and state [1–5]. Biomarkers can be employed in clinics to predict, diagnose, and monitor the disease by examining changes in the level of proteins in disease groups and normal groups. These biomarkers are broadly divided into three groups: physical measurements or phenotypes such as brain imaging, extracellular beta-amyloid (Aβ) plaque deposition [6,7]; DNA-based biomarkers [5,8]; and protein biomarkers [9–12]. Proteomics has undoubtedly garnered more attention recently, greatly attributed to the fact that proteins are abundant in body fluids and are more stable than DNA and metabolites. A unique biomarker for a particular disease should accurately reflect the progression of that disease therefore providing a better prognosis for early diagnosis. Hundreds of millions of dollars have been spent each year for the past several years on research to discover protein biomarkers of many human diseases. In the year 2013 alone, there were over 17,000 publications related to the keyword “biomarker” in Web of Science. Even with this level of investment and research effort, the US Food and Drug Administration (FDA) approves no more than 2 new biomarkers for plasma per year [13,14]. This very low research to market rate is a very significant challenge [15].

Alzheimer’s disease slowly and pathologically develops from preclinical phase or an early phase into a fully expressed clinical syndrome, hence there is a serious need for biomarkers that reflect core elements of Alzheimer’s disease (AD) for the purposes of early diagnosis. The primary biomarkers currently employed for AD diagnosis are measurements of Aβ and tau [16–18].

**Figure 1.**
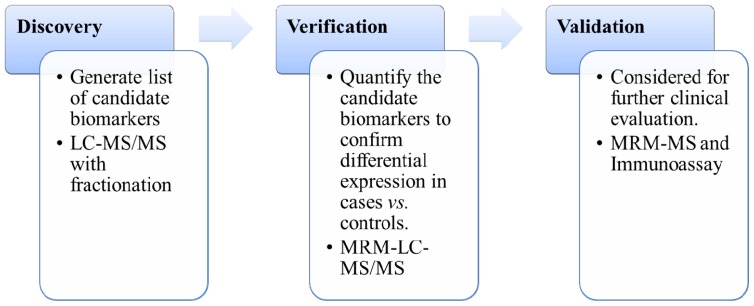
Categorization of the biomarker development. LC-MS/MS, liquid chromatography tandem mass spectrometry. MRM-LC-MS/MS: multiple reaction monitoring-liquid chromatography tandem mass spectrometry. MRM-MS: multiple reaction monitoring-mass spectrometry.

There are generally three different stages in the development of new biomarkers (Figure 1): The discovery phase, the verification phase, and the validation phase [2,13,19]. First, proteomics technologies are applied to blood (plasma or serum) or cerebral spinal fluid (CSF) to identify more than a million biomarker candidates. Second, biomarker candidates are quantified in a limited number (10–100 thousands) of clinical samples to confirm differential expression in blood\CSF from cases *vs.* controls. Finally, beyond verification phase, clinical validation requires large-scale cases to existing clinical tests [2]. The development of proteomics has been driven by the growth of new technologies for peptides/protein fractionation, mass spectrometry instruments, labeling reagents, and bioinformatic tools. A critical goal of mass spectrometry-based (MS-based) proteomics is to identify and quantify proteins from biological samples. Recent advances in MS-based proteomics provide indispensable tools in the clinic. This review will provide an overview of biomarkers for AD. It will focus on discovery technologies by using examples of biomarker discovery with MS-based proteomics technologies, and discuss potential ways to identify additional biomarkers.

## 2. Sources of Biomarkers

Proteomics on human samples has mainly focused on available biological fluids such as blood (plasma or serum), CSF, urine, and saliva. For biomarkers applications, a single biomarker is probably insufficient for the accurate representation of a disease. Therefore, multiple biomarker profiles need to be identified in different types of, DNA, RNA, microRNA (miRNA), and protein, including modifications from DNA, gene, and post-translational modification (PTM) proteins.

### 2.1. DNA-Based Biomarkers

DNA methylation studies have shown strong potential for biomarker identification [20–22]. RNA can be obtained from cells and it is also present in exosomes in plasma. The complexity of RNA has only recently begun to be realized [23,24]. miRNA, key players of post-transcriptional gene regulation, are approximately 20 nucleotides long non-coding RNA. An estimated 70% of miRNAs are expressed in the brain [25]. They can be detected using methods such as real-time polymerase chain reaction (RT-PCR), and microarrays through deep sequencing technologies.

One study [26] describing a search for miRNA abundance in the hippocampal region of AD patients’ brain, show upregulation of miR-9, -125b, and -128 compared to age-matched controls. Furthermore, miR-34a [27], -145b, and -155 [28,29] are significantly higher in abundance compared to age-matched controls in CSF and extracellular fluid (ECF).

MiR-107 was shown to be downregulated in AD [30]. The levels of these miRNAs were also reduced in AD patients, and include miR-137, -181c, -9, -29a, -29b [31], and -146a [25].

There is evidence showing that the changes at miRNA levels are associated with some parts of AD pathology, such as in the case of miR-16 which could potentially inhibit expression of amyloid precursor protein (APP) in age-related senescence-accelerated mouse prone 8 (SAMP8) mice [32]. Several differently expressed miRNA in AD were identified, but these results have not yet been confirmed [25,33–35]. There is still progress to be made in continually monitoring the changes in the level of individual miRNA as biomarkers for AD [31,36–38].

### 2.2. Blood-Based Biomarkers

The blood proteome is one of the most complex components of the human proteome [7,39]. With about 60–80 g/L protein content in blood plasma, the concentration of protein is extraordinarily higher than 0.15–0.45 g/L in CSF [40]. As a source of biomarkers, several blood biomarkers candidates have been proposed [9,11,41]. Blood is in contact with all cells of the organism, and (1) it is easily accessible and represents a non-invasive liquid biopsy; (2) it provides a cost and time efficient way to clinical trials. Blood can be separated into different components: plasma and serum. Serum is similar to plasma in composition but without the clotting factor [42].

As we are aware, Aβ is a widely researched plasma biomarker for AD. However, it is unclear the extent to which blood Aβ levels accurately reflect the presence or state of AD. Koyama [43] searched prospective studies published between 1995 and 2011 regarding Aβ_40_, Aβ_42_, and Aβ_42_:Aβ_40_. The literature showed lower Aβ_42_:Aβ_40_ ratios were mainly associated with AD and dementia.

There are several biomarkers identified in blood in last decades, such as Apolipoprotein E (ApoE) localized on chromosome 19. The report from Gupta [44] showed that the levels of plasma ApoE in AD revealed an obvious relationship between ApoE levels and AD. Apo A-IV as an up-regulated protein was identified in serum samples of AD [45]. Interleukins (IL-1α, IL-6) is one of the strongest evidence of inflammatory agents that increase the risk of AD [46]. Clusterin (CLU) is a lipoprotein found to be part of amyloid plaques. Two studies have identified variants in CLU is associated with the risks of AD [47,48]. α-1-antichymotrypsin (α-ACT) participates in the inflammatory cascade of AD and enhances the formation of amyloid fibrils. Several reports showed increased concentrations of α-ACT in serum of AD patients [49–51]. Activity-dependent neuroprotective protein (ADNP) is expressed primarily in the cerebellum, hippocampus, and cerebral cortex in human brain [52,53]. However, when Yang [45] tested serum samples from 45 early-stage AD patients; their results showed ADNP was down-regulated.

The likelihood of blood samples having more influence on biomarker discovery is high, despite being a relatively, disadvantaged source, peripheral blood is a complex tissue containing too many proteins, making it non-specific.

### 2.3. CSF-Based Biomarkers

As an alternative to blood, other biofluids such as urine and cerebrospinal fluid (CSF) can be used in biomarker discovery [54–56]. The CSF, being most proximal to the Central Nervous System (CNS), (1) directly interacts with the space of the brain and reflects biochemical changes that occur in the brain; (2) CSF collection is a highly invasive procedure that may cause patient discomfort and immediate side effects [57]. Normally, CSF contains components from both the blood and the CNS [58,59], which also plays an ideal role for identifying biomarkers for AD.

CSF biomarkers are used more commonly for diagnosing AD in the clinic. According to Blennow [9], CSF biomarkers for AD can be divided into two groups: the basic one and the core one.

Albumin is a basic group protein. Test results show that albumin can be produced by the liver [60] and it makes up more than 50% of the protein content in blood. Due to fact that most of the albumin present in CSF is blood-derived, the ratio of CSF to serum albumin concentrations is a biomarker for blood-brain barrier (BBB) function. An increase in this ratio indicates BBB damage, which is normal in AD patients, unless the patient has concomitant cerebrovascular disease [61].

Secretory Ca^2+^-dependent phospholipases A2 (sPLA2) is another BBB factor. Chalbot [62,63] demonstrated that in AD patients, the sPLA2 level is elevated compared to the control group, making it a good biomarker for BBB function.

Visinin-like 1(VLP-1) is expressed in neurons. The levels of VLP-1 are increased in CSF after stroke in rats and the same regulation was present in serum after stroke in humans [64,65].

Microtubule-associated protein tau (TAU) was first reported in 1975 [66]. One study showed that almost all samples have shown an increase in T-tau in AD patients by approximately 300% with a sensitivity and specificity of 80%–90% [67]. High T-tau level in CSF has also been associated with fast progression from mild cognitive impairment (MCI) to AD [68], and with rapid cognitive decline and a high mortality rate in patients with AD [69,70]. High CSF phosphorylated-tau (P-tau) has the same correlations; there is a strong relationship between MCI and AD [68], and with rapid cognitive decline in AD [69].

Neurofilament proteins (NF) are specifically found in neurons [71], an uptrend level of NF in CSF reflects axonal damage. High levels of the neurofilament heavy chain isoform NFHSM135 have been found in AD [72].

Fatty acid binding protein 3 (FABP3) are a family of small intracellular proteins that facilitate the transport of fatty acids. Lower levels of FABP3 have been detected in brains from AD [73]. Chromogranin A (CHGA) was also shown to be present in neurons. Lower levels of CHGA [74] and unchanged levels of CHGA and CHGB have been found in the CSF of AD patients [75]. Neurogranin (NRGN) was first isolated from bovine brain. Reduced protein levels of NRGN have been observed in post mortem brains from patients with AD [76,77]. S100 calcium-binding protein beta (S-100B) is composed of two β subunits. CSF levels of S-100B are elevated in patients with AD [78–80]. Glial fibrillary acidic protein (GFAP) is the main astrocytic intermediate filament protein. CSF levels of GFAP are also increased in AD [80].

Several studies have investigated the various analytes in AD related to Aβ, such as β-site APP cleaving enzyme 1 (BACE1), sAPPα/sAPPβ, or Aβ oligomers for their possibility as biomarkers. Recently, a large amount of information regarding BACE1 in blood or CSF has been available [81–83], except quantitative analysis by MS-based proteomics. Despite the fact that previous studies strongly support BACE1 as a potential biomarker for AD, quantitative information is required to be part of the analysis and evidence support.

## 3. Technologies for Proteomic Analysis

**Figure 2.**
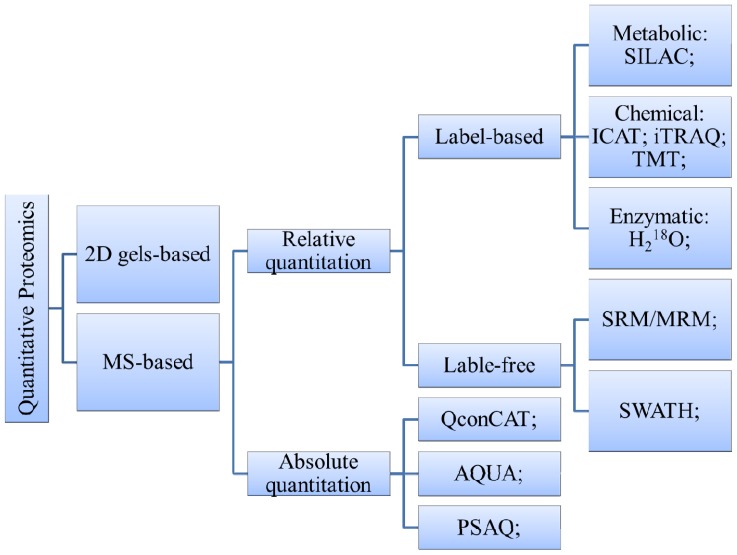
Overview of main quantitative proteomics methods [19]. 2D gels: Two-dimensional gel electrophoresis; MS: mass spectrometry; SILAC: Stable Isotope Labeling by Amino Acids in Cell Culture; ICAT: Isotope-Coded Affinity Tags; iTRAQ: isobaric Tag for Relative and Absolute Quantitation; TMT: Tandem Mass Tags; SRM/MRM: selected/multiple reaction monitoring; QconCAT: concatenated peptides; AQUA: Absolute Quantitation; PSAQ: Protein Standard Absolute Quantification; SWATH: Sequential Isolation Windows.

In recent years, proteomics has emerged as a viable approach used not only to identify novel diagnostic and therapeutic biomarkers, but also to research clinical diagnostics and drug development for AD [84]. Due to the complexity of biological systems and proteins, proteomics strategy and methods are tested to be more effective and a better fit for the goal of protein identification and quantification (Figure 2). Such a coordinated method typically includes several components: sample preparation, primary separation, protein or peptide identification and bioinformatic data analysis.

### 3.1. Biomarkers Discovery

#### 3.1.1. Two-Dimensional Polyacrylamide Gel Electrophoresis (2D-Gel) Based Method

Depending on the level of content in proteins, especially high-abundance proteins like albumin, the analysis of low abundance proteins is more difficult than it is in CSF. On the other hand, the enormous amounts of lipids and salts in blood plasma are also obstacles when using proteomic methods. Therefore, the pre-analytical handling of samples needs special attention.

The classic proteomics platform, a 2D-gel, is an effective approach for quantification and separation of complex protein mixtures. With a 2D-gel, it is possible to simultaneously detect and quantify up to a few thousand proteins using the same gel. The final result will consist of a protein map in which different proteins are separated into spots; protein spots are then subjected to analysis using MS [85]. Under the condition of using narrow range pH gels, 2D-gel can resolve more than 5000 proteins [86].

Using 2D-gel and liquid chromatography tandem mass spectrometry (LC-MS/MS), Liao [87] identified six potential plasma biomarkers, some of these molecules are known to play important roles in CNS microglia activation, such as α-1-antitrypsin (AAT). However, this procedure presents some shortcomings. Proteins present at low abundance in blood cannot be detected because they are concealed under highly abundant molecules [88]. The same approach was also applied by Hye [89]; they identified a large number of potential AD markers and also a histone, mainly from the complement and immunoglobulin system. Zhang [90] employed multi-dimensional LC in combination with 1D- and 2D-gels to detect serum-based biomarkers in AD. Using matrix-assisted laser desorption/ionization-quadrupole-time of flight (MALDI-QTOF) and Ion-Trap-MS, they identified several proteins increased in AD, including inflammatory response mediators, complement factor H, complement components C3 and C4, and α-2-macroglobulin.

Thambisetty [91–93] performed an experiment, which first identified seven plasma proteins, then validated five of them, complement components C3 and C3a, complement factor-I, fibrinogen gamma chain, and α-1microglobulin. Henkel [94] removed 12 high-abundance proteins via anion exchange and reversed phase-LC (RPLC). The resulting LC fractions were analyzed on a 2D-gel. They detected 20 significant differentially expressed proteins by MS analysis, some known to be involved in the pathophysiology of AD.

#### 3.1.2. MS-Based Method

Another quantitative proteomics strategy is MS-based approaches. To some extent, 2D-gel quantification has largely been replaced by MS-based methods. In MS-based phase, proteins are not separated prior to digestion, and then peptides are fractionated via LC. There are two portions in MS-based proteomics: stable isotope labeling and label-free approaches [95–99]. Protein or peptide labeling using chemical isobaric tags, include Isotope-Coded Affinity Tags (ICAT), isobaric Tag for Relative and Absolute Quantitation (iTRAQ), Tandem Mass Tags (TMT); Enzymatic labeling includes H_2_^18^O; Metabolic labeling, such as stable isotope labeling by amino acids in cell culture (SILAC).

Jing [100] employed a micro LC-MS combined with ICAT labeling, they identified more than 300 proteins in AD, 13 of which showed changes with advancing age. In another study, Fu [101] used ICAT to analyze mitochondrial fractions from knock-in mice. They found 46 proteins with altered expressions, 32 increased and 14 decreased. Choe [102] used an 8-plex version of an isobaric reagent mixed with CSF proteins from AD, the results showed a number of protein expression changes.

Multiple reaction monitoring-mass spectrometry (MRM-MS) combined with isotope-labeled QconCAT peptides has been successfully applied to quantify proteins [103,104]. Chen [105] performed this technique on severe AD cases, and set up a quantitative method to assess expression levels of clusterin. Later, they also developed this protocol for quantification of total APP and APP695 [106].

### 3.2. Biomarkers Verification and Validation

Once a list of prospective biomarkers has been identified in the identification section, a validation test should be followed with the goal of selecting one with extraordinary potential for clinical diagnosis from the biomarkers candidate menu. This portion needs to employ a clinical strategy and be tested on thousands of samples that directly reflect the clinical population. It will take years for MS-based methods to complete this experiment cycle, from the emergence advanced into targeted proteomics stage. Biomarker candidates are usually identified via the later strategy, but it was not able to accomplish the union with high measurement accuracy and precision [2,107]. Therefore, setting up a supplementary analytical system to validate potential markers is highly recommended. Immune-based assays are frequently considered ideal for validation and development for clinical diagnosis with their highly sensitive methods, including Western blotting, enzyme-linked immunosorbent assay (ELISA), and radioimmunoassay. They provide a greater performance of sensitivity compared with LC-MS, and are immediately accessible in research and development settings of the clinical laboratory [108–110].

ELISA is usually preferred to radioisotope use,, though a multiplicity of variables are still challenging, because ELISA can be seriously influenced by the avidity and dilution of capture and detection antibodies, incubation time, temperature and concentration, and enzyme and substrate types [2]. In a previous study, only 30% of the markers have been validated with commercially available antibodies with respect to both protein identification and quantification [111,112]. This is due to that first of all, some antibodies are nonspecific, and second of all, there are arguments regarding protein confirmation results between Western blot and MS-based proteomics [113].

Numerous reports have demonstrated that MS-based methods can be robust and accurate. The reasoning is as follows: the absence of commercially available antibodies, present technical issues, high costs related to the development of high-quality antibodies [110], and many proteins show the state with PTM, e.g., glycosylation of ceruloplasmin, which means modified peptides are typically not taken into account [114].

Based on the reasons mentioned above, several MS-based technologies have been employed for validation phase [110] as an alternative. MRM-MS is typically employed for biomarker quantitation and validation. It is an MS/MS mode unique to triple quadrupole (QQQ) MS instruments. MRM can enhance the lower detection limit for peptides due to its ability to rapidly and continuously monitor exclusively for the specific ions of interest. Furthermore, MRM analysis combined with stable isotope also offers multiplexing capability and increases the reliability of quantification [115]. Pannee [116] reported on a matrix effect-resistant method for the measurement of the Aβ42, together with Aβ40 and Aβ38 in human CSF using MRM-MS. They detected Aβ42 at a lower limit of quantification of 62.5 pg/mL and coefficients of variations below 10%. These outstanding benefits have expanded the potential applications of MRM quantitation beyond biomarker validation and into the phase of biomarker identification.

## 4. Conclusions and Future Perspectives

Currently, “-omics” research mainly focus on the identification, qualification, and application of diagnostic and prognostic biomarkers. The latest advances in “-omics” technologies have improved our understanding of the development and biology of AD.

### 4.1. Peptidomics

The far-reaching changes in proteomics have brought new technologists into the fields of protein biomarker discovery and clinical chemistry. As multidisciplinary fields grow, more analytical techniques are being used to study biological issues. “Peptidomics” is the study of bioactive peptides, endogenous peptides, and small proteins found in biological samples with the aim to understand all information of peptides and small proteins in a biological system [117,118]. In biological fluids, these peptides are active with biological function in normal and disease states; represent protein synthesis, processing, and degradation [119,120]. Some bioactive peptides that have been identified in CSF and plasma, such as VGF peptide (*m*/*z* 4807) were found to be obviously altered in CSF of AD [121–123]. Furthermore, some bioactive peptides from proteins that were digested aberrantly could be valuable not only for disease diagnosis, but also for explaining the mechanisms involved in the disease [110].

### 4.2. Modification-Specific Proteomics

PTM of proteins, such as phosphorylation, ubiquitination and glycosylation, play key biological roles in function, activity, localization, and interaction. Aberrant modifications have now been recognized as an attribute of many neurodegenerative diseases [124]. Ando [125] suggested that Pin1 protein is strongly linked to the tau pathology, and such changes in Pin1 posttranslational modification (phosphorylation, *N*-acetylation, and oxidation) may also represent interesting biomarkers to AD.

Due to relatively low level of PTM, an approach of detecting PTM uses MS-based needs enrichment method, such as immobilized metal ion affinity chromatography (IMAC), TiO_2_ As with any technology, the one being utilized to find potential biomarkers of neurodegenerative disease, has its shortcomings and limitations [126,127]. It must be emphasized, however, that it is unlikely that there will be specific antibodies available commercially against proteins with novel PTM soon, so MS-based approaches will likely still be viewed as the golden technology in validating the candidate proteins with PTM for the next few years.

### 4.3. Metabonomics

Another “-omics” technology for biomarker projects is metabonomics. It is considered as one of the fastest developing workflows in biomarker research. The profile of small-molecular-weight substances present on a range of different sample types including cells, tissue and body fluids are known as the metabolites. Nuclear magnetic resonance (NMR) is a particularly powerful tool for metabolite structural test. An MS-based approach is a sensitive one to identify and quantify in complex biological systems. Both of these methods have become analytical standards in metabonomics studies [128,129]. Kiyoshi [130] used NMR-based metabolomics of transgenic AD mice model; they suggested that levels of three small molecules were significantly upregulated compared to control mice. HPLC-MS was performance on blood plasma from AD patients, with results that potential biomarkers were identified in lysophosphatidylcholine, sphingosine and tryptophan [131]. The number of studies in the metabolic area continues to expand [132,133], and it will hopefully lead us to new biomarkers.

As AD is multifactorial, no single biomarker will be able to explain its progression and pathology. The success of -omics technologies has made it possible to collect high-density biological readouts related to physiological and pathological processes. Combining these, results offers a systems biology approach which can enhance our understanding of biochemical insight in the organism. Their use has thus forced a rapid change from lab-based study to clinic-style investigations.

### 4.4. Conclusions

Biomarkers have been examined can play a critical role in diagnostics and drug development. The research for AD biomarker has taken many directions, includes measuring in blood, CSF and urine. Previous studies to use proteomics to discovery better biomarkers have developed for decades, however, these attempts have met less success samples which was used to be a clinical biomarker for AD. The challenge to validate a clinical biomarker because of many factors: the complexity of the body fluids, low abundance protein biomarkers must be detected, there are many steps critical for biomarker from discovery to validate. Despite the challenges exist, there are some “-omics” areas leading us into the future study for AD biomarker, such as peptidomics, modification-specific proteomics and metabonomics. A combined all of these “-omics” approach reveals new insight to study biomarker.

## References

[1] Zhang X., Li L., Wei D., Yap Y., Chen F. (2007). Moving cancer diagnostics from bench to bedside. Trends Biotechnol.

[2] Rifai N., Gillette M.A., Carr S.A. (2006). Protein biomarker discovery and validation: The long and uncertain path to clinical utility. Nat. Biotechnol.

[3] Poste G. (2011). Bring on the biomarkers. Nature.

[4] Puntmann V.O. (2009). How to guide on biomarkers: Biomarker definitions, Validation and applications with examples from cardiovascular disease. Postgrad. Med. J.

[5] Anderson D.C., Kodukula K. (2014). Biomarkers in pharmacology and drug discovery. Biochem. Pharmacol.

[6] Ballard C., Gauthier S., Corbett A., Brayne C., Aarsland D., Jones E. (2011). Alzheimer’s disease. Lancet.

[7] Ghidoni R., Paterlini A., Benussi L. (2013). Translational proteomics in Alzheimer’s disease and related disorders. Clin. Biochem.

[8] Podlesniy P., Figueiro-Silva J., Llado A., Antonell A., Sanchez-Valle R., Alcolea D., Lleo A., Molinuevo J.L., Serra N., Trullas R. (2013). Low cerebrospinal fluid concentration of mitochondrial DNA in preclinical alzheimer disease. Ann. Neurol.

[9] Blennow K., Hampel H., Weiner M., Zetterberg H. (2010). Cerebrospinal fluid and plasma biomarkers in alzheimer disease. Nat. Rev. Neurol.

[10] Hampel H., Blennow K., Shaw L.M., Hoessler Y.C., Zetterberg H., Trojanowski J.Q. (2010). Total and phosphorylated tau protein as biological markers of Alzheimer’s disease. Exp. Gerontol.

[11] Trojanowski J.Q., Vandeerstichele H., Korecka M., Clark C.M., Aisen P.S., Petersen R.C., Blennow K., Soares H., Simon A., Lewczuk P. (2010). Update on the biomarker core of the Alzheimer’s disease neuroimaging initiative subjects. Alzheimer’s Dement.

[12] Weiner M.W., Veitch D.P., Aisen P.S., Beckett L.A., Cairns N.J., Green R.C., Harvey D., Jack C.R., Jagust W., Liu E. (2012). The Alzheimer’s disease neuroimaging initiative: A review of papers published since its inception. Alzheimer’s Dement.

[13] Whiteaker J.R., Lin C., Kennedy J., Hou L., Trute M., Sokal I., Yan P., Schoenherr R.M., Zhao L., Voytovich U.J. (2011). A targeted proteomics–based pipeline for verification of biomarkers in plasma. Nat. Biotechnol.

[14] Anderson N.L. (2010). The clinical plasma proteome: A survey of clinical assays for proteins in plasma and serum. Clin. Chem.

[15] Nature editorial office (2010). Valid concerns. The reporting of candidate biomarkers for disease must be rigorous to drive translational research. Nature.

[16] Prvulovic D., Hampel H. (2011). Amyloid β (Aβ) and phospho-tau (p-tau) as diagnostic biomarkers in Alzheimer’s disease. Clin. Chem. Lab. Med.

[17] Watt A.D., Perez K.A., Faux N.G., Pike K.E., Rowe C.C., Bourgeat P., Salvado O., Masters C.L., Villemagne V.L., Barnham K.J. (2011). Increasing the predictive accuracy of amyloid-beta blood-borne biomarkers in Alzheimer’s disease. J. Alzheimer’s Dis.

[18] Rosenmann H. (2012). CSF biomarkers for amyloid and tau pathology in Alzheimer’s disease. J. Mol. Neurosci.

[19] Zhang Y., Fonslow B.R., Shan B., Baek M.C., Yates J.R. (2013). Protein analysis by shotgun/bottom-up proteomics. Chem. Rev.

[20] Ramaswamy S., Perou C.M. (2003). DNA microarrays in breast cancer: The promise of personalised medicine. Lancet.

[21] Simpson R.J., Lim J.W., Moritz R.L., Mathivanan S. (2009). Exosomes: Proteomic insights and diagnostic potential. Expert Rev. Proteomics.

[22] Kit H.A., Nielsen H.M., Tost J. (2012). DNA methylation based biomarkers: Practical considerations and applications. Biochimie.

[23] Thambisetty M., Lovestone S. (2010). Blood-based biomarkers of Alzheimer’s disease: Challenging but feasible. Biomark. Med.

[24] Hunter M.P., Ismail N., Zhang X., Aguda B.D., Lee E.J., Yu L., Xiao T., Schafer J., Lee M.L., Schmittgen T.D. (2008). Detection of microrna expression in human peripheral blood microvesicles. PLoS One.

[25] Muller M., Kuiperij H.B., Claassen J.A., Kusters B., Verbeek M.M. (2014). MicroRNAs in Alzheimer’s disease: Differential expression in hippocampus and cell-free cerebrospinal fluid. Neurobiol. Aging.

[26] Lukiw W.J. (2007). Micro–RNA speciation in fetal, adult and Alzheimer’s disease hippocampus. Neuroreport.

[27] Wang X., Liu P., Zhu H., Xu Y., Ma C., Dai X., Huang L., Liu Y., Zhang L., Qin C. (2009). MiR-34a, a microRNA up-regulated in a double transgenic mouse model of Alzheimer’s disease, inhibits bcl2 translation. Brain Res. Bull.

[28] Lukiw W.J., Alexandrov P.N., Zhao Y., Hill J.M., Bhattacharjee S. (2012). Spreading of Alzheimer’s disease inflammatory signaling through soluble micro-RNA. Neuroreport.

[29] Alexandrov P.N., Dua P., Hill J.M., Bhattacharjee S., Zhao Y., Lukiw W.J. (2012). MicroRNA (miRNA) speciation in Alzheimer’s disease (AD) cerebrospinal fluid (CSF) and extracellular fluid (ECF). Int. J. Biochem. Mol. Biol.

[30] Wang W.X., Rajeev B.W., Stromberg A.J., Ren N., Tang G., Huang Q., Rigoutsos I., Nelson P.T. (2008). The expression of microRNA MIR-107 decreases early in Alzheimer’s disease and may accelerate disease progression through regulation of β-site amyloid precursor protein-cleaving enzyme 1. J. Neurosci.

[31] Geekiyanage H., Jicha G.A., Nelson P.T., Chan C. (2012). Blood serum miRNA: Non-invasive biomarkers for Alzheimer’s disease. Exp. Neurol.

[32] Liu W., Liu C., Zhu J., Shu P., Yin B., Gong Y., Qiang B., Yuan J., Peng X. (2012). MicroRNA-16 targets amyloid precursor protein to potentially modulate alzheimer’s-associated pathogenesis in samp8 mice. Neurobiol. Aging.

[33] Cogswell J.P., Ward J., Taylor I.A., Waters M., Shi Y., Cannon B., Kelnar K., Kemppainen J., Brown D., Chen C. (2008). Identification of miRNA changes in Alzheimer’s disease brain and CSF yields putative biomarkers and insights into disease pathways. J. Alzheimer’s Dis.

[34] Schonrock N., Matamales M., Ittner L.M., Gotz J. (2012). MicroRNA networks surrounding APP and amyloid-β metabolism-implications for Alzheimer’s disease. Exp. Neurol.

[35] Tan L., Yu J.T., Hu N. (2013). Non-coding RNAs in Alzheimer’s disease. Mol. Neurobiol.

[36] Sheinerman K.S., Tsivinsky V.G., Abdullah L., Crawford F., Umansky S.R. (2013). Plasma microRNA biomarkers for detection of mild cognitive impairment: Biomarker validation study. Aging.

[37] Sheinerman K.S., Tsivinsky V.G., Crawford F., Mullan M.J., Abdullah L., Umansky S.R. (2012). Plasma microRNA biomarkers for detection of mild cognitive impairment. Aging.

[38] Kiko T., Nakagawa K., Tsuduki T., Furukawa K., Arai H., Miyazawa T. (2014). MicroRNAs in plasma and cerebrospinal fluid as potential markers for Alzheimer’s disease. J. Alzheimer’s Dis.

[39] Thadikkaran L., Siegenthaler M.A., Crettaz D., Queloz P.A., Schneider P., Tissot J.D. (2005). Recent advances in blood-related proteomics. Proteomics.

[40] Zürbig P., Jahn H. (2012). Use of proteomic methods in the analysis of human body fluids in Alzheimer research. Electrophoresis.

[41] Mayeux R., Schupf N. (2011). Blood-based biomarkers for Alzheimer’s disease: Plasma Aβ40 and Aβ42, and genetic variants. Neurobiol. Aging.

[42] Issaq H.J., Xiao Z., Veenstra T.D. (2007). Serum and plasma proteomics. Chem. Rev.

[43] Koyama A., Okereke O.I., Yang T., Blacker D., Selkoe D.J., Grodstein F. (2012). Plasma amyloid-β as a predictor of dementia and cognitive decline: A systematic review and meta-analysis. Arch. Neurol.

[44] Gupta V.B., Laws S.M., Villemagne V.L., Ames D., Bush A.I., Ellis K.A., Lui J.K., Masters C., Rowe C.C., Szoeke C. (2011). Plasma apolipoprotein E and alzheimer disease risk. Neurology.

[45] Yang M.H., Yang Y.H., Lu C.Y., Jong S.B., Chen L.J., Lin Y.F., Wu S.J., Chu P.Y., Chung T.W., Tyan Y.C. (2012). Activity-dependent neuroprotector homeobox protein: A candidate protein identified in serum as diagnostic biomarker for Alzheimer’s disease. J. Proteomics.

[46] Du Y., Dodel R.C., Eastwood B.J., Bales K.R., Gao F., Lohmuller F., Muller U., Kurz A., Zimmer R., Evans R.M. (2000). Association of an interleukin 1α polymorphism with Alzheimer’s disease. Neurology.

[47] Adams S., Harold A., Bremner W., Bhatti A. (2009). Immediate post-parathyroidectomy stridor resolved with intravenous calcium. BMJ Case Rep.

[48] Lambert J.C., Heath S., Even G., Campion D., Sleegers K., Hiltunen M., Combarros O., Zelenika D., Bullido M.J., Tavernier B. (2009). Genome-wide association study identifies variants at CLU and PICALM associated with Alzheimer’s disease. Nat. Genet.

[49] Oishi M., Mochizuki Y., Yoshihashi H., Takasu T., Nakano E. (1996). Laboratory examinations correlated with severity of dementia. Ann. Clin. Lab. Sci.

[50] Karsidag T., Tuzun S., Kemik A.S., Purisa S., Unlu A. (2012). Alpha-1 protease inhibitor and antichymotrypsin levels in acute pancreatitis. Turkish J. Trauma Emerg. Surg.

[51] Guan F., Gu J., Hu F., Zhu Y., Wang W. (2012). Association between α1-antichymotrypsin signal peptide-15A/T polymorphism and the risk of Alzheimer’s disease: A meta-analysis. Mol. Biol. Rep.

[52] Zamostiano R., Pinhasov A., Gelber E., Steingart R.A., Seroussi E., Giladi E., Bassan M., Wollman Y., Eyre H.J., Mulley J.C. (2001). Cloning and characterization of the human activity-dependent neuroprotective protein. J. Biol. Chem.

[53] Fernandez-Montesinos R., Torres M., Baglietto-Vargas D., Gutierrez A., Gozes I., Vitorica J., Pozo D. (2010). Activity-dependent neuroprotective protein (ADNP) expression in the amyloid precursor protein/presenilin 1 mouse model of Alzheimer’s disease. J. Mol. Neurosci.

[54] Shaw L.M., Korecka M., Clark C.M., Lee V.M., Trojanowski J.Q. (2007). Biomarkers of neurodegeneration for diagnosis and monitoring therapeutics. Nat. Rev. Drug Discov.

[55] Sonnen J.A., Keene C.D., Montine K.S., Li G., Peskind E.R., Zhang J., Montine T.J. (2007). Biomarkers for Alzheimer’s disease. Expert Rev. Neurother.

[56] Clark C.M., Davatzikos C., Borthakur A., Newberg A., Leight S., Lee V.M., Trojanowski J.Q. (2008). Biomarkers for early detection of Alzheimer pathology. Neurosignals.

[57] De Almeida S.M., Shumaker S.D., LeBlanc S.K., Delaney P., Marquie-Beck J., Ueland S., Alexander T., Ellis R.J. (2011). Incidence of post-dural puncture headache in research volunteers. Headache.

[58] Oreskovic D., Klarica M. (2010). The formation of cerebrospinal fluid: Nearly a hundred years of interpretations and misinterpretations. Brain Res. Rev.

[59] Kroksveen A.C., Opsahl J.A., Aye T.T., Ulvik R.J., Berven F.S. (2011). Proteomics of human cerebrospinal fluid: Discovery and verification of biomarker candidates in neurodegenerative diseases using quantitative proteomics. J. Proteomics.

[60] Miller L.L., Bale W.F. (1954). Synthesis of all plasma protein fractions except gamma globulins by the liver; The use of zone electrophoresis and lysine-epsilon-C14 to define the plasma proteins synthesized by the isolated perfused liver. J. Exp. Med.

[61] Blennow K., Wallin A., Fredman P., Karlsson I., Gottfries C.G., Svennerholm L. (1990). Blood-brain barrier disturbance in patients with Alzheimer’s disease is related to vascular factors. Acta Neurol. Scand.

[62] Chalbot S., Zetterberg H., Blennow K., Fladby T., Grundke-Iqbal I., Iqbal K. (2009). Cerebrospinal fluid secretory Ca^2+^-Dependent phospholipase A2 activity is increased in alzheimer disease. Clin. Chem.

[63] Chalbot S., Zetterberg H., Blennow K., Fladby T., Grundke-Iqbal I., Iqbal K. (2010). Cerebrospinal fluid secretory Ca^2+^-dependent phospholipase A2 activity: A biomarker of blood-cerebrospinal fluid barrier permeability. Neurosci. Lett.

[64] Laterza O.F., Modur V.R., Crimmins D.L., Olander J.V., Landt Y., Lee J.M., Ladenson J.H. (2006). Identification of novel brain biomarkers. Clin. Chem.

[65] Lee J.M., Blennow K., Andreasen N., Laterza O., Modur V., Olander J., Gao F., Ohlendorf M., Ladenson J.H. (2008). The brain injury biomarker VLP-1 is increased in the cerebrospinal fluid of Alzheimer disease patients. Clin. Chem.

[66] Weingarten M.D., Lockwood A.H., Hwo S.Y., Kirschner M.W. (1975). A protein factor essential for microtubule assembly. Proc. Natl. Acad. Sci. USA.

[67] Blennow K., Zetterberg H. (2009). Cerebrospinal fluid biomarkers for Alzheimer’s disease. J. Alzheimer’s Dis.

[68] Blom E.S., Giedraitis V., Zetterberg H., Fukumoto H., Blennow K., Hyman B.T., Irizarry M.C., Wahlund L.O., Lannfelt L., Ingelsson M. (2009). Rapid progression from mild cognitive impairment to Alzheimer’s disease in subjects with elevated levels of tau in cerebrospinal fluid and the APOE epsilon4/epsilon4 genotype. Dement. Geriatr. Cogn. Disord.

[69] Samgard K., Zetterberg H., Blennow K., Hansson O., Minthon L., Londos E. (2010). Cerebrospinal fluid total tau as a marker of Alzheimer’s disease intensity. Int. J. Geriatr. Psychiatry.

[70] Wallin A.K., Hansson O., Blennow K., Londos E., Minthon L. (2009). Can CSF biomarkers or pre-treatment progression rate predict response to cholinesterase inhibitor treatment in Alzheimer’s disease?. Int. J. Geriatr. Psychiatry.

[71] Liu Q., Xie F., Siedlak S.L., Nunomura A., Honda K., Moreira P.I., Zhua X., Smith M.A., Perry G. (2004). Neurofilament proteins in neurodegenerative diseases. Cell Mol. Life Sci.

[72] Brettschneider J., Petzold A., Schottle D., Claus A., Riepe M., Tumani H. (2006). The neurofilament heavy chain (NFH) in the cerebrospinal fluid diagnosis of Alzheimer’s disease. Dement. Geriatr. Cogn. Disord.

[73] Cheon M.S., Kim S.H., Fountoulakis M., Lubec G. (2003). Heart type fatty acid binding protein (H-FABP) is decreased in brains of patients with Down syndrome and Alzheimer’s disease. J. Neural. Transm. Suppl.

[74] Perrin R.J., Craig-Schapiro R., Malone J.P., Shah A.R., Gilmore P., Davis A.E., Roe C.M., Peskind E.R., Li G., Galasko D.R. (2011). Identification and validation of novel cerebrospinal fluid biomarkers for staging early Alzheimer’s disease. PLoS One.

[75] Mattsson N., Savman K., Osterlundh G., Blennow K., Zetterberg H. (2010). Converging molecular pathways in human neural development and degeneration. Neurosci. Res.

[76] Davidsson P., Blennow K. (1998). Neurochemical dissection of synaptic pathology in Alzheimer’s disease. Int. Psychogeriatr.

[77] Thorsell A., Bjerke M., Gobom J., Brunhage E., Vanmechelen E., Andreasen N., Hansson O., Minthon L., Zetterberg H., Blennow K. (2010). Neurogranin in cerebrospinal fluid as a marker of synaptic degeneration in Alzheimer’s disease. Brain Res.

[78] Herrmann M., Ebert A.D., Galazky I., Wunderlich M.T., Kunz W.S., Huth C. (2000). Neurobehavioral outcome prediction after cardiac surgery: Role of neurobiochemical markers of damage to neuronal and glial brain tissue. Stroke.

[79] Peskind E.R., Griffin W.S., Akama K.T., Raskind M.A., van Eldik L.J. (2001). Cerebrospinal fluid S-100B is elevated in the earlier stages of Alzheimer’s disease. Neurochem. Int.

[80] Jesse S., Steinacker P., Cepek L., von Arnim C.A., Tumani H., Lehnert S., Kretzschmar H.A., Baier M., Otto M. (2009). Glial fibrillary acidic protein and protein S-100B: Different concentration pattern of glial proteins in cerebrospinal fluid of patients with Alzheimer’s disease and Creutzfeldt-Jakob disease. J. Alzheimer’s Dis.

[81] Wang H., Li R., Shen Y. (2013). Beta-secretase: Its biology as a therapeutic target in diseases. Trends Pharmacol. Sci.

[82] Snyder H.M., Carrillo M.C., Grodstein F., Henriksen K., Jeromin A., Lovestone S., Mielke M.M., O’Bryant S., Sarasa M., Sjogren M. (2014). Developing novel blood-based biomarkers for Alzheimer’s disease. Alzheimer’s Dement.

[83] Hilpert H., Guba W., Woltering T.J., Wostl W., Pinard E., Mauser H., Mayweg A.V., Rogers-Evans M., Humm R., Krummenacher D. (2013). βsecretase (BACE1) inhibitors with high *in vivo* efficacy suitable for clinical evaluation in Alzheimer’s disease. J. Med. Chem.

[84] Altelaar A.F., Munoz J., Heck A.J. (2013). Next-generation proteomics: Towards an integrative view of proteome dynamics. Nat. Rev. Genet.

[85] Chevallet M., Luche S., Diemer H., Strub J.M., van Dorsselaer A., Rabilloud T. (2008). Sweet silver: A formaldehyde-free silver staining using aldoses as developing agents, with enhanced compatibility with mass spectrometry. Proteomics.

[86] Fey S.J., Larsen P.M. (2001). 2D or not 2D. Two-dimensional gel electrophoresis. Curr. Opin. Chem. Biol.

[87] Song F., Poljak A., Smythe G.A., Sachdev P. (2009). Plasma biomarkers for mild cognitive impairment and Alzheimer’s disease. Brain Res. Rev.

[88] Apweiler R., Aslanidis C., Deufel T., Gerstner A., Hansen J., Hochstrasser D., Kellner R., Kubicek M., Lottspeich F., Maser E. (2009). Approaching clinical proteomics: Current state and future fields of application in fluid proteomics. Clin. Chem. Lab. Med.

[89] Hye A., Lynham S., Thambisetty M., Causevic M., Campbell J., Byers H.L., Hooper C., Rijsdijk F., Tabrizi S.J., Banner S. (2006). Proteome-based plasma biomarkers for Alzheimer’s disease. Brain.

[90] Zhang R., Barker L., Pinchev D., Marshall J., Rasamoelisolo M., Smith C., Kupchak P., Kireeva I., Ingratta L., Jackowski G. (2004). Mining biomarkers in human sera using proteomic tools. Proteomics.

[91] Thambisetty M., Hye A., Foy C., Daly E., Glover A., Cooper A., Simmons A., Murphy D., Lovestone S. (2008). Proteome-based identification of plasma proteins associated with hippocampal metabolism in early Alzheimer’s disease. J. Neurol.

[92] Thambisetty M., Simmons A., Hye A., Campbell J., Westman E., Zhang Y., Wahlund L.O., Kinsey A., Causevic M., Killick R. (2011). Plasma biomarkers of brain atrophy in Alzheimer’s disease. PLoS One.

[93] Thambisetty M., An Y., Kinsey A., Koka D., Saleem M., Guntert A., Kraut M., Ferrucci L., Davatzikos C., Lovestone S. (2012). Plasma clusterin concentration is associated with longitudinal brain atrophy in mild cognitive impairment. Neuroimage.

[94] Henkel A.W., Muller K., Lewczuk P., Muller T., Marcus K., Kornhuber J., Wiltfang J. (2012). Multidimensional plasma protein separation technique for identification of potential Alzheimer’s disease plasma biomarkers: A pilot study. J. Neural Transm.

[95] Buts K., Michielssens S., Hertog M.L., Hayakawa E., Cordewener J., America A.H., Nicolai B.M., Carpentier S.C. (2014). Improving the identification rate of data independent label-free quantitative proteomics experiments on non-model crops: A case study on apple fruit. J. Proteomics.

[96] Cole L.M., Bluff J.E., Carolan V.A., Paley M.N., Tozer G.M., Clench M.R. (2014). MALDI-MSI and label free LC-ESI-MS/MS shotgun proteomics to investigate protein induction in a murine fibrosarcoma model following treatment with a vascular disrupting agent. Proteomics.

[97] Mohayeji M., Capriotti A.L., Cavaliere C., Piovesana S., Samperi R., Stampachiacchiere S., Toorchi M., Lagana A. (2014). Heterosis profile of sunflower leaves: A label free proteomics approach. J. Proteomics.

[98] Hakimi A., Auluck J., Jones G.D., Ng L.L., Jones D.J. (2014). Assessment of reproducibility in depletion and enrichment workflows for plasma proteomics using label-free quantitative data-independent LC–MS. Proteomics.

[99] Liu X., Hu Y., Pai P.J., Chen D., Lam H. (2014). Label-free quantitative proteomics analysis of antibiotic response in staphylococcus aureus to oxacillin. J. Proteome Res.

[100] Zhang J., Goodlett D.R., Quinn J.F., Peskind E., Kaye J.A., Zhou Y., Pan C., Yi E., Eng J., Wang Q. (2005). Quantitative proteomics of cerebrospinal fluid from patients with alzheimer disease. J. Alzheimer’s Dis.

[101] Fu Y.J., Xiong S., Lovell M.A., Lynn B.C. (2009). Quantitative proteomic analysis of mitochondria in aging PS-1 transgenic mice. Cell Mol. Neurobiol.

[102] Choe L., D’Ascenzo M., Relkin N.R., Pappin D., Ross P., Williamson B., Guertin S., Pribil P., Lee K.H. (2007). 8-plex quantitation of changes in cerebrospinal fluid protein expression in subjects undergoing intravenous immunoglobulin treatment for Alzheimer’s disease. Proteomics.

[103] Beynon R.J., Doherty M.K., Pratt J.M., Gaskell S.J. (2005). Multiplexed absolute quantification in proteomics using artificial QCAT proteins of concatenated signature peptides. Nat. Methods.

[104] Ding C., Li Y., Kim B.J., Malovannaya A., Jung S.Y., Wang Y., Qin J. (2011). Quantitative analysis of cohesin complex stoichiometry and SMC3 modification-dependent protein interactions. J. Proteome Res.

[105] Chen J., Wang M., Turko I.V. (2012). Mass spectrometry quantification of clusterin in the human brain. Mol. Neurodegener.

[106] Chen J., Wang M., Turko I.V. (2013). Quantification of amyloid precursor protein isoforms using quantification concatamer internal standard. Anal. Chem.

[107] Kingsmore S.F. (2006). Multiplexed protein measurement: Technologies and applications of protein and antibody arrays. Nat. Rev. Drug Discov.

[108] Vitzthum F., Behrens F., Anderson N.L., Shaw J.H. (2005). Proteomics: From basic research to diagnostic application. A review of requirements & needs. J. Proteome Res.

[109] Lista S., Faltraco F., Prvulovic D., Hampel H. (2013). Blood and plasma-based proteomic biomarker research in Alzheimer’s disease. Prog. Neurobiol.

[110] Wei X., Li L. (2009). Mass spectrometry-based proteomics and peptidomics for biomarker discovery in neurodegenerative diseases. Int. J. Clin. Exp. Pathol.

[111] Khorvash F., Abdi F., Dialami K., Kooshki A.M. (2011). Can serum procalcitonin and C-reactive protein as nosocomial infection markers in hospitalized patients without localizing signs?. J. Res. Med. Sci.

[112] Abdi F., Quinn J.F., Jankovic J., McIntosh M., Leverenz J.B., Peskind E., Nixon R., Nutt J., Chung K., Zabetian C. (2006). Detection of biomarkers with a multiplex quantitative proteomic platform in cerebrospinal fluid of patients with neurodegenerative disorders. J. Alzheimer’s Dis.

[113] Aebersold R., Burlingame A.L., Bradshaw R.A. (2013). Western blots *versus* selected reaction monitoring assays: Time to turn the tables?. Mol. Cell Proteomics.

[114] Shi M., Caudle W.M., Zhang J. (2009). Biomarker discovery in neurodegenerative diseases: A proteomic approach. Neurobiol. Dis.

[115] Zhang K., Schrag M., Crofton A., Trivedi R., Vinters H., Kirsch W. (2012). Targeted proteomics for quantification of histone acetylation in Alzheimer’s disease. Proteomics.

[116] Pannee J., Portelius E., Oppermann M., Atkins A., Hornshaw M., Zegers I., Hojrup P., Minthon L., Hansson O., Zetterberg H. (2013). A selected reaction monitoring (SRM)-based method for absolute quantification of Aβ38, Aβ40, and Aβ42 in cerebrospinal fluid of Alzheimer’s disease patients and healthy controls. J. Alzheimer’s Dis.

[117] Menschaert G., Vandekerckhove T.T., Baggerman G., Schoofs L., Luyten W., van Criekinge W. (2010). Peptidomics coming of age: A review of contributions from a bioinformatics angle. J. Proteome Res.

[118] Tinoco A.D., Saghatelian A. (2011). Investigating endogenous peptides and peptidases using peptidomics. Biochemistry.

[119] Monacelli F., Borghi R., Pacini D., Serrati C., Traverso N., Odetti P. (2014). Pentosidine determination in CSF: A potential biomarker of alzheimer’disease?. Clin. Chem. Lab. Med.

[120] Kingwell K. (2013). Alzheimer disease: CSF levels of mitochondrial DNA—A new biomarker for preclinical alzheimer disease?. Nat. Rev. Neurol.

[121] Ertekin-Taner N. (2013). Alzheimer disease: The quest for Alzheimer disease genes—Focus on CSF tau. Nat. Rev. Neurol.

[122] Schmidt C., Artjomova S., Hoeschel M., Zerr I. (2013). CSF prion protein concentration and cognition in patients with Alzheimer disease. Prion.

[123] Wijte D., McDonnell L.A., Balog C.I., Bossers K., Deelder A.M., Swaab D.F., Verhaagen J., Mayboroda O.A. (2012). A novel peptidomics approach to detect markers of Alzheimer’s disease in cerebrospinal fluid. Methods.

[124] Ballatore C., Lee V.M.-Y., Trojanowski J.Q. (2007). Tau-mediated neurodegeneration in Alzheimer’s disease and related disorders. Nat. Rev. Neurosci.

[125] Ando K., Dourlen P., Sambo A.V., Bretteville A., Belarbi K., Vingtdeux V., Eddarkaoui S., Drobecq H., Ghestem A., Begard S. (2013). Tau pathology modulates PIN1 post-translational modifications and may be relevant as biomarker. Neurobiol. Aging.

[126] Opii W.O., Joshi G., Head E., Milgram N.W., Muggenburg B.A., Klein J.B., Pierce W.M., Cotman C.W., Butterfield D.A. (2008). Proteomic identification of brain proteins in the canine model of human aging following a long-term treatment with antioxidants and a program of behavioral enrichment: Relevance to Alzheimer’s disease. Neurobiol. Aging.

[127] Zhang J. (2007). Proteomics of human cerebrospinal fluid—The good, the bad, and the ugly. PROTEOMICS-Clin. Appl.

[128] Deja S., Barg E., Mlynarz P., Basiak A., Willak-Janc E. (2013). 1H NMR-based metabolomics studies of urine reveal differences between type 1 diabetic patients with high and low HbAc1 values. J Pharm. Biomed. Anal.

[129] Huang Y., Tian Y., Li G., Li Y., Yin X., Peng C., Xu F., Zhang Z. (2013). Discovery of safety biomarkers for realgar in rat urine using UFLC-IT-TOF/MS and 1H NMR based metabolomics. Anal. Bioanal. Chem.

[130] Fukuhara K., Ohno A., Ota Y., Senoo Y., Maekawa K., Okuda H., Kurihara M., Okuno A., Niida S., Saito Y. (2013). NMR-based metabolomics of urine in a mouse model of Alzheimer’s disease: Identification of oxidative stress biomarkers. J. Clin. Biochem. Nutr.

[131] Li N.J., Liu W.T., Li W., Li S.Q., Chen X.H., Bi K.S., He P. (2010). Plasma metabolic profiling of Alzheimer’s disease by liquid chromatography/mass spectrometry. Clin. Biochem.

[132] Wishart D.S., Knox C., Guo A.C., Eisner R., Young N., Gautam B., Hau D.D., Psychogios N., Dong E., Bouatra S. (2009). Hmdb: A knowledgebase for the human metabolome. Nucleic Acids Res.

[133] Gika H.G., Theodoridis G.A., Plumb R.S., Wilson I.D. (2014). Current practice of liquid chromatography-mass spectrometry in metabolomics and metabonomics. J. Pharm. Biomed. Anal.

